# Machine learning at the edge for AI-enabled multiplexed pathogen detection

**DOI:** 10.1038/s41598-023-31694-6

**Published:** 2023-03-23

**Authors:** Vahid Ganjalizadeh, Gopikrishnan G. Meena, Matthew A. Stott, Aaron R. Hawkins, Holger Schmidt

**Affiliations:** 1 School of Engineering, University of California, Santa Cruz, 1156 High Street, Santa Cruz, CA 95064 USA; 2 Electrical and Computer Engineering Department, Brigham Young University, Provo, UT 84602 USA

**Keywords:** Electrical and electronic engineering, Integrated optics, Computer science

## Abstract

Multiplexed detection of biomarkers in real-time is crucial for sensitive and accurate diagnosis at the point of use. This scenario poses tremendous challenges for detection and identification of signals of varying shape and quality at the edge of the signal-to-noise limit. Here, we demonstrate a robust target identification scheme that utilizes a Deep Neural Network (DNN) for multiplex detection of single particles and molecular biomarkers. The model combines fast wavelet particle detection with Short-Time Fourier Transform analysis, followed by DNN identification on an AI-specific edge device (Google Coral Dev board). The approach is validated using multi-spot optical excitation of Klebsiella Pneumoniae bacterial nucleic acids flowing through an optofluidic waveguide chip that produces fluorescence signals of varying amplitude, duration, and quality. Amplification-free 3× multiplexing in real-time is demonstrated with excellent specificity, sensitivity, and a classification accuracy of 99.8%. These results show that a minimalistic DNN design optimized for mobile devices provides a robust framework for accurate pathogen detection using compact, low-cost diagnostic devices.

## Introduction

**Figure 1:**
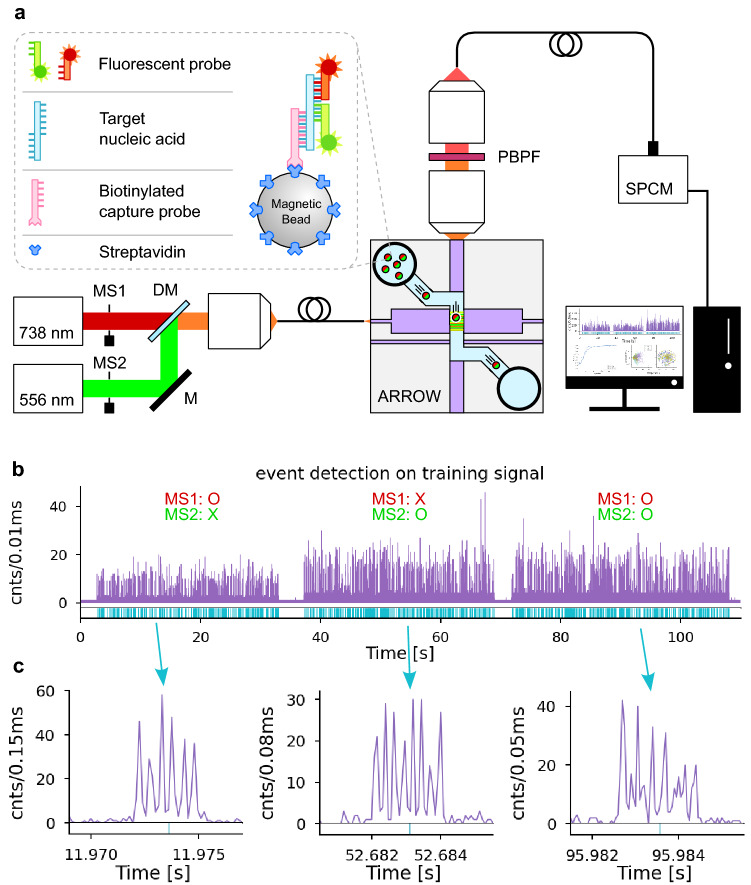
Experimental setup and dataset preparation. (**a**) Fluorescence signals are collected from labeled Klebsiella Pneumoniae bacteria captured on microbeads by a target-specific sandwich assay. Inset shows the sandwich design of the fluorescence probes, which consists of both green and red dyes. The sample is prepared off-chip and flows into the ARROW chip using negative pressure-driven flow. The multimode-interference (MMI) pattern formed at the MMI WG-analyte channel intersection is wavelength dependent and defines the signatures of different signals. The experiment is done in three steps depending on if beads are excited by the red laser, green laser, or both colors. Combinations of mechanical shutters (MS1 and MS2) are used to create the three mentioned stages. Fluorescence signals are collected off-chip through a penta-bandpass-filter (PBPF) to remove excitation photons. Emission photons are then detected and recorded on a PC by a single-photon-counting-module. (**b**) The recorded data trace (training signal) is analyzed by the PCWA program to detect events. Events are cropped and saved for dataset preparation. Locations of detected events are shown with blue markers. Event detection and cropping are done automatically. **c** Example events from three groups of events.

Detection and identification of biomolecules are essential parts of diagnostic devices in the disease control realm. The COVID-19 pandemic has accelerated the use of home testing for early detection and repeated monitoring, and it can be expected that point-of-care analysis will expand in volume and capabilities. Some of the challenges here are sample preparation and fluid handling, sensitivity and specificity, data acquisition and processing, portability, and connectivity^1,2^. Various micro and nanotechnologies have resulted in sensors applicable to on-chip molecular diagnostics. For example, a complementary metal–oxide–semiconductor (CMOS) based biosensor utilizing a nanomechanical membrane-bridge design^3^ was introduced to measure the concentration of a phenytoin drug in a liquid sample with a microelectromechanical (MEMS) approach. Paper-based analytical devices (PADs) are another good example of point-of-care diagnostics. They have evolved over the past decade due to properties such as low cost, biocompatibility, simple design and usage, and flexibility in pursuing affordable disposable testing units^4–6^. While sample analysis can be done in a few minutes, these devices have a relatively high limit of detection (LOD > µM), demanding amplification and cultivation time (usually several hours) for highly sensitive nucleic acid analysis. Among amplification-based techniques, quantitative polymerase chain reaction (qPCR) is still the gold standard method in most of laboratory test instruments due to its very high sensitivity (~ 100 copies/mL) and versatility^7–9^. A newer single-tube technique with a relatively inexpensive and simple amplification process called loop-mediated-isothermal-amplification (LAMP) has become more popular in the past decade^10,11^. Further assay simplification based on elimination of amplification steps and chip-scale integration may be desirable in some applications. In the single-molecule sensitivity regime, nanopore devices as electrical biosensors have shown promise as label-free and amplification-free ultra-high sensitivity diagnostic tools^12,13^. Fluorescence-based single-molecule detection sensitivity was demonstrated in optofluidic waveguide devices, enabling amplification-free detection of Ebola virus (EBOV) RNAs with low limits of detection (LoD) down to 90 aM^14,15^. Multiplex analysis was implemented on these single-molecule optofluidic ARROW sensors by creating spectrally and/or spatially dependent multi-spot excitation patterns at the output of multi-mode interference (MMI) waveguides^16,17^. The spatial multi-spot pattern translates into a temporal multi-peak signal (see Fig. 1c) which can be easily identified by signal processing algorithms that recognize the characteristic time difference ∆*t* between fluorescence peaks from a single target. Up to 7× multiplexed detection of nucleic acids has been demonstrated using spectrally, spatially, and velocity-dependent translation of the excitation pattern into time-dependent multi-peak fluorescence signals^17,18^. Using deliberately patterned signals has been used in other contexts as well. For example, telecom-like signal encoding was implemented in cell cytometry using electrical resistive pulse sensing in which the pattern of events generated by flowing cells depends on the arrangement of the electrodes and decodes digital signals from different channels with a very high accuracy^19–21^. A multi-finger electrode electrical biosensor was studied to demonstrate the effect of increased electrode count in the impedance signal-to-noise ratio (SNR)^22–25^. Encoding signals in biosensing applications can take advantage of more complicated information coding techniques such as multiplexing, error correction, and identification^23,26,27^ with a recent trend toward machine learning techniques^28^. Optimizing the signal analysis method of choice is critically important as real-world device imperfections tend to compromise signal quality and, thus, the reliability of the signal analysis. In the case of an optofluidic MMI waveguide device, these can include fabrication-induced variations in MMI excitation patterns, velocity variations due to fluidic dynamics of flowing targets, and signal-to-noise ratio variations caused by a range of fluorescence signal intensities. These types of nonidealities are added on top of the signal quality limitations faced by point-of-care devices, where components should be produced at low cost, and environmental factors often impact signal quality. These intrinsic challenges can be alleviated by a powerful signal analysis approach that can operate in real-time.

Recently, we have introduced a new parallel cluster wavelet analysis (PCWA) approach that can identify events in a time series, such as fluorescence from single molecules, with high accuracy and speed^29^. However, the analysis speed and accuracy drop for multiplexed detection due to the need to tune the custom wavelet functions for different targets before analysis, which adds extra overhead to the chip-to-chip variation of fluorescence signal characteristics. Augmenting PCWA with Machine Learning (ML) can solve this problem and lead to robust, multiplexed diagnostics.

Machine learning and artificial intelligence (AI) ideas and theories have been around for many decades, if not a century, with the model of nonlinear neurons forming a network back in the mid-1990s^30^ and became a functional tool in pattern recognition with better learning theories being developed in late 1990s^31–33^. Even though the ML concept is not new, the field has exploded in the last 2 decades when large-scale high-performance computation became possible to handle ever-larger amounts of data. AI has become a part of our daily life and is used in numerous areas, including agriculture, environmental studies, autonomous vehicles, economics, marketing, drug development, entertainment, and medical diagnosis^34–43^. In biomedical diagnosis, artificial intelligence-assisted methods at different levels, from large-scale population data down to sensory data analysis, have been developed, underscoring the advantages of novel machine-learning over classical techniques^44–46^.

Here, we introduce a full-stack AI-assisted framework integrated with a multiplexed detection platform to detect and identify biomolecules. Its capabilities are demonstrated on an optofluidic MMI waveguide device for multiplexed detection of fluorescently tagged biomolecules^16^. The system is evaluated by analysis of three combinatorial multi-color signals from representative plasmids of drug-resistant Klebsiella Pneumoniae bacteria. Compared to previously used techniques, outstanding recall and accuracy are observed. Running in real-time, the detection framework was able to discover 93.8% of the events, with 99.8% classification accuracy. An affordable portable AI-specific development board (Google Coral Dev board) was then selected to show the compatibility of the optofluidic diagnostic system with the state-of-the-art processing schemes^47^. The Edge-TPU (tensor processing unit) available in this development board is a coprocessor capable of performing four tera-operations per second (TOPS) with a power consumption of 500 mW/TOPS^48^. Finally, we evaluated the timing performance and real-time sample analysis capability of the framework. This system will open new avenues for ultra-fast point-of-care devices for diagnostic and other applications.

## Results

### Experimental setup and event detection

Our framework can be applied to any time-series signal that features different time signatures for different targets. We introduce it using a fluorescence detection assay for Klebsiella Pneumoniae bacteria nucleic acid biomarkers. A sandwich design assay binds target DNA strands to a pulldown sequence on a streptavidin-coated microbead, after which they are labeled with fluorescent probes in two colors (Fig. 1a). The assay is, therefore, sensitive to both red and green excitation wavelengths, allowing for three possible fluorescence signals (red only, green only, and red + green), depending on the excitation wavelengths used. Targets are flowed through and detected on an ARROW-based optofluidic as shown in Fig. 1a. The sensitivity (limit of detection) of this flow-based optofluidic fluorescence assay is 90 aM^15^. Two lasers running at 738 nm and 556 nm are coupled into a single-mode optical fiber, and two mechanical shutters, MS1 and MS2, are placed in the light paths to toggle colors on and off independently. The experiment is done in three stages, first with only 738 nm excitation (MS1 = Open, MS2 = Close), second with only 556 nm (MS1 = Close, MS2 = Open), and third with both 738 nm and 556 nm excitations active simultaneously (MS1 = Open, MS2 = Open). The sample containing the fluorescent targets is introduced into the chip by applying negative pressure to one of the reservoirs and then introducing the sample into the other reservoir using a pipette. The recorded fluorescence time trace from the chip is stored on a desktop computer (Fig. 1b). The multi-mode interference excitation waveguide produces different spot patterns for the two wavelengths in the intersecting fluidic channel with the number of spots *N* determined by Eq. (1)

1 $$N \times \lambda = \frac{{n_{C} w^{2} }}{L}$$

where λ is the respective input wavelength, *w* is the effective waveguide width, *n*_*C*_ is the refractive index *n*_*C,*_ and *L* is the length of the MMI waveguide^49^. These spatial excitation patterns are transformed into the time domain as particles flow through the channel at a given velocity and fluorescence. Events from the first stage have six peaks, events from the second stage have eight peaks, and events from the third stage contain both 6 and 8 peaks. We named these events by splitting the word KPneu into two parts (KPn for six peaks and eu for eight peaks) to represent which probe has been excited in the detected event and will use these labels throughout the article. This approach effectively creates a 3× multiplex assay with three distinct signal shapes corresponding to the excitation wavelength selections. Figure 1c shows example events for each stage which illustrate the different fluorescence patterns and the nonidealities present in this configuration. These include finite background signal, peak-to-peak amplitude variations due to fabrication imperfections, and different peak-to-peak spacing Δ*t* due to velocity variations from pressure fluctuations and position in the channel. A coefficient of variation of 44.85% is observed in the velocity distribution of 1544 detected particles. Minimizing these nonidealities would require raising the production cost to increase the precision of the microfabrication processes. Some nonidealities, such as cross-section fluid flow speed variations, are simply due to the nature of the microfluidic channels being used. Point-of-care devices, meant to be low-cost and compact, must always deal with signal imperfections. A natural strategy is, therefore, to adapt to these imperfections by employing machine learning to recognize the signal pattern for a given device and detect and identify fluorescence events with good sensitivity and accuracy.

The first step in our signal-recognition process is to detect events from the recorded fluorescence signal using a fast multi-scale continuous wavelet-based technique called PCWA^29^ with multi-spot-Gaussian (MSG) basis functions to match the characteristic signals for events in each stage (see Supplementary Fig. S1a). After locating events in the time trace, a collection of cropped windows comprising 1024 data points centered on an individual event’s time location is used to create the dataset for neural network analysis and classification. False identified or overlapping events are removed from the dataset using a multi-factor signal quality check to improve the neural network model’s training (see Supplementary Fig. S2). The entire event detection, cropping, filtering, and annotation is automated and does not require any user involvement.

### Dataset preparation and deep learning

**Figure 2:**
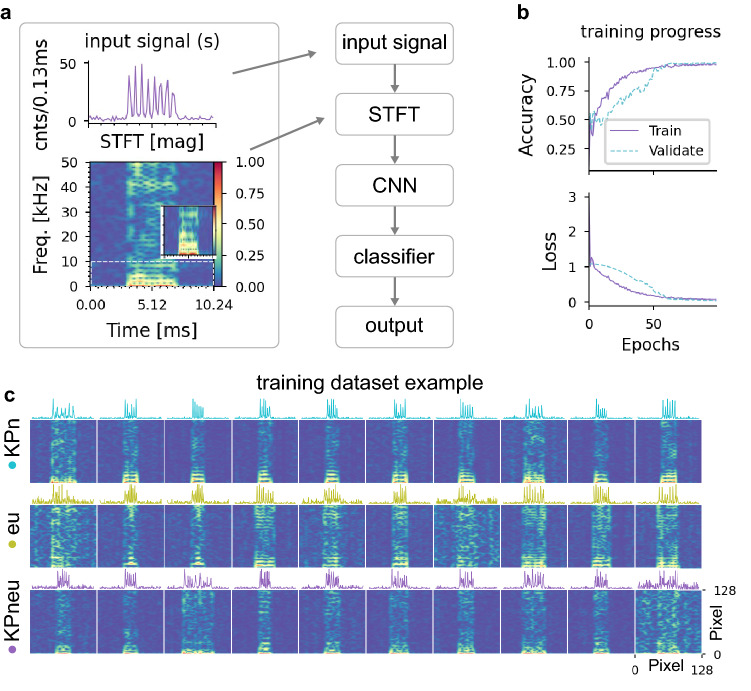
Deep Neural Network and dataset. (**a**) DNN model consists of two conv2d layers followed by a max_pooling2d and a batch normalization in between. A classifier layer (dense) maps extracted features from previous layers to the output classes. The plot on the left shows how the input signal is first converted into a 128 × 128 pixels spectrogram using STFT. (**b**) The progress of training with accuracy and loss metrics evolves over 100 epochs. Training is stopped at the 100th epoch to prevent overfitting. After 90 epochs, the loss value stays below 0.1. **c** Examples of events from the training dataset. Spectrograms and the corresponding cropped time events are shown together, but only the spectrograms and labels of the events are fed to the DNN model during training.

The main components of the deep neural network are shown in (Fig. 2a, right). Fluorescence events with temporal signatures representing the MMI pattern exhibit unique time–frequency features for each class of events which also depend on the velocity of the flowing particles. Therefore, we take advantage of a short-time-Fourier-transform (STFT), which has been used in speech, EEG, and ECG data to extract time–frequency features^50–53^. The input signal is first transformed into a 128 × 128 pixel image expressing the magnitude of the STFT of the event (Fig. 2a, left). A DNN model is designed, built, and tested from scratch for this application with the goal of minimal complexity and high compatibility with the target inferencing edge platform. First, the input image is down-sampled into a 64 × 64 pixel image. Two cascaded convolutional layers are deployed to extract a pool of features. Finally, a dense layer with a softmax activation function is used to classify input images into three output classes corresponding to red, green, and red-green fluorescence. The model contains only 6454 parameters (6436 trainable) which is orders of magnitude lower than popular image classification models^54^. The model is a small application-specific model adapted from the LeNet CNN model, which was used to recognize handwritten digits^55^. The DNN model is trained using a supervised learning technique by a dataset of spectrograms calculated for events detected from the training time trace (see Fig. 2c). Since labeling can be easily and automatically done based on the lasers’ ‘on’ and ‘off’ states, supervised learning was the best-suited learning technique here.

Training was done by minimizing the categorical cross-entropy loss function *L*, defined by Eq. (2). To minimize the loss, a stochastic gradient descent (SGD) method was applied by using Adam optimizer^56^ with the default learning parameters (learning rate = 0.001, *β*_1_ = 0.9, *β*_2_ = 0.999, *ε* = 10^−7^) set by TensorFlow.

2 $$L = - \frac{1}{N}\sum\limits_{i = 0}^{N - 1} {\sum\limits_{c = 0}^{2} {y_{i,c} \log \left( {p_{i,c} } \right)} }$$

where *p*_*i,c*_ is the probability value of class *c*, for sample *i*, returned by a softmax layer of the classifier. *y*_*i,c*_ is the true (one-hot) label of each class for sample *i*, and *N* = 90 is the size of minibatch used during training. The softmax function is defined by Eq. (3):

3 $$p_{c} = \sigma (x_{c} ) = \frac{{e^{{x_{c} }} }}{{\sum\limits_{c = 0}^{2} {e^{{x_{c} }} } }}$$

After 90 epochs, the loss value drops below 0.1, and we stopped at 100 epochs to prevent overfitting (Fig. 2b). We found that a ~ 250 nL volume of sample (approximate concentration 10^6^ beads/mL) contains enough events (~ 300 events/class) to train the model and the entire training step takes less than 15 min on a single desktop machine.

### DNN classifier and transfer learning

**Figure 3:**
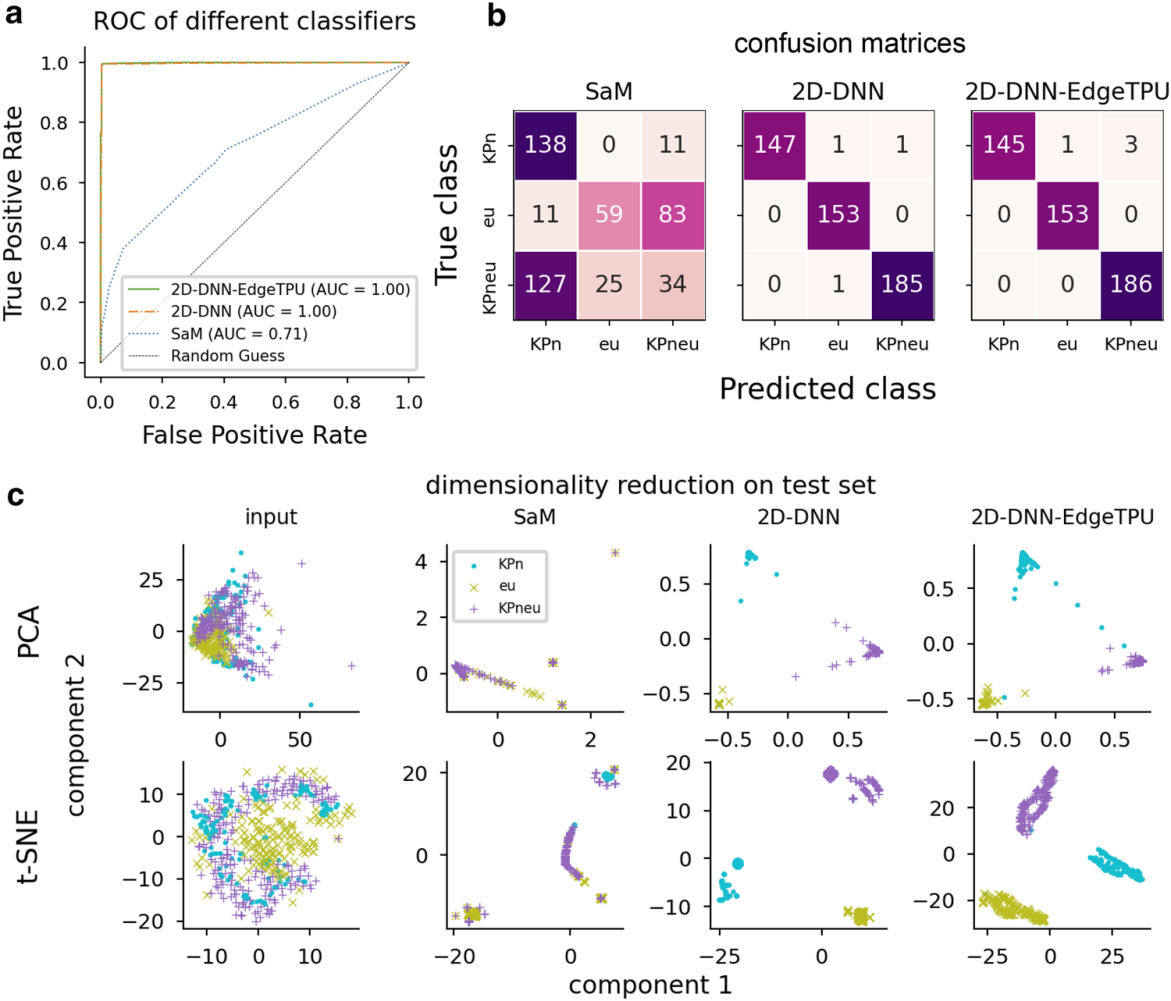
Performance comparison. (**a**) ROC plot for the studied classifiers. The DNN model, on a PC (2D-DNN) or Coral (2D-DNN-EdgeTPU), outperforms the previously used unsupervised classifier, SaM. (**b**) Confusion matrices for different classifiers highlight only 3 and 4 events misclassified for the 2D-DNN and 2D-DNN-EdgeTPU models, respectively. SaM fails to classify eu and KPneu events due to their low signal-to-noise (SNR) characteristics. (**c**) Dimensionality reduction applied to input and outputs of different classifiers is made using PCA and t-SNE. Input data fails to form clusters in linear (PCA) and nonlinear (t-SNE) methods. SaM, conventionally used for MMI event classification, weakly classifies events from different classes. The proposed 2D-DNN model shows robust classification performance noticeable in both PCA and t-SNE scatter plots with clearly separated clusters. The transferred 2D-DNN model running on the Edge device with 8-bit integer quantization performs comparably to the 32-bit float model of 2D-DNN.

A Google Coral Dev Board^57^ is selected as the AI accelerator hardware for the system. It incorporates a system-on-module (SoM) tensor processing unit (Edge-TPU) chip alongside an ARM-based microcomputer with Raspberry Pi form factor and essential connectivity modules^58^. Currently, this device is limited to only inferencing, meaning that the training step needs to be done on other platforms (i.e., desktop, cloud). The model is implemented in TensorFlow^59^, which has the best compatibility with the Coral board. The trained DNN model (2D-DNN) on a desktop computer (32-floating point) is converted into a quantized 8-bit integer model compatible with Edge-TPU (2D-DNN-EdgeTPU) via a transfer-learning procedure^60^. A test dataset from a test experiment is created the same way as the training dataset to assure that none of the classifiers had been exposed to test events. Performance evaluation (Fig. 3a) shows the receiver’s operating characteristic curve (ROC) for the 2D-DNN as well as the conventionally used classifier for the multiplexed fluorescence detection, shift-and-multiply (SaM). The ROC curve visualizes the true positive and false-positive rates, i.e., sensitivity and specificity, for a given selection threshold. The 2D-DNN model outperforms SaM with a near-perfect area-under-curve (AUC ≅ 1) and only a few false identified events visualized in the confusion matrices (Fig. 3b). This demonstrates that our approach is able to correctly find and classify fluorescence signals from single particles. This is the first principal result of this article.

Dimensionality reduction, as a prevalent tool for data visualization, especially in high dimensional data, helps to look for a relationship or meaningful structural information in an understandable lower-dimensional space^61^. Principle component analysis (PCA) as the most common linear and t-distributed stochastic neighbor embedding (t-SNE) as the other popular nonlinear dimensionality reduction techniques are used here to compare how each classifier model is able to group events into clusters^61–65^. Both PCA and t-SNE fail to find structural information and form clusters when applied to the raw input signals (Fig. 3c, left column). At the same time, SaM can separate some of the events when visualized by nonlinear t-SNE embedding. 2D-DNN (both versions) maps high dimensional input signals into a linearly and non-linearly separable three-dimension space (Fig. 3c). There is a negligible drop in 2D-DNN-EdgeTPU classifier performance related to the precision drop from the transfer-learning step. The average inference time on the Coral board using ARMx64 CPU is 4.14 ms, while a 2 × speedup is observed by utilizing the Edge-TPU accelerator resulting in a 2.07 ms average inference time. The inference is done on events with a 10.24 ms window size, indicating real-time classification performance.

### Real-time pathogen detection on the edge

**Figure 4:**
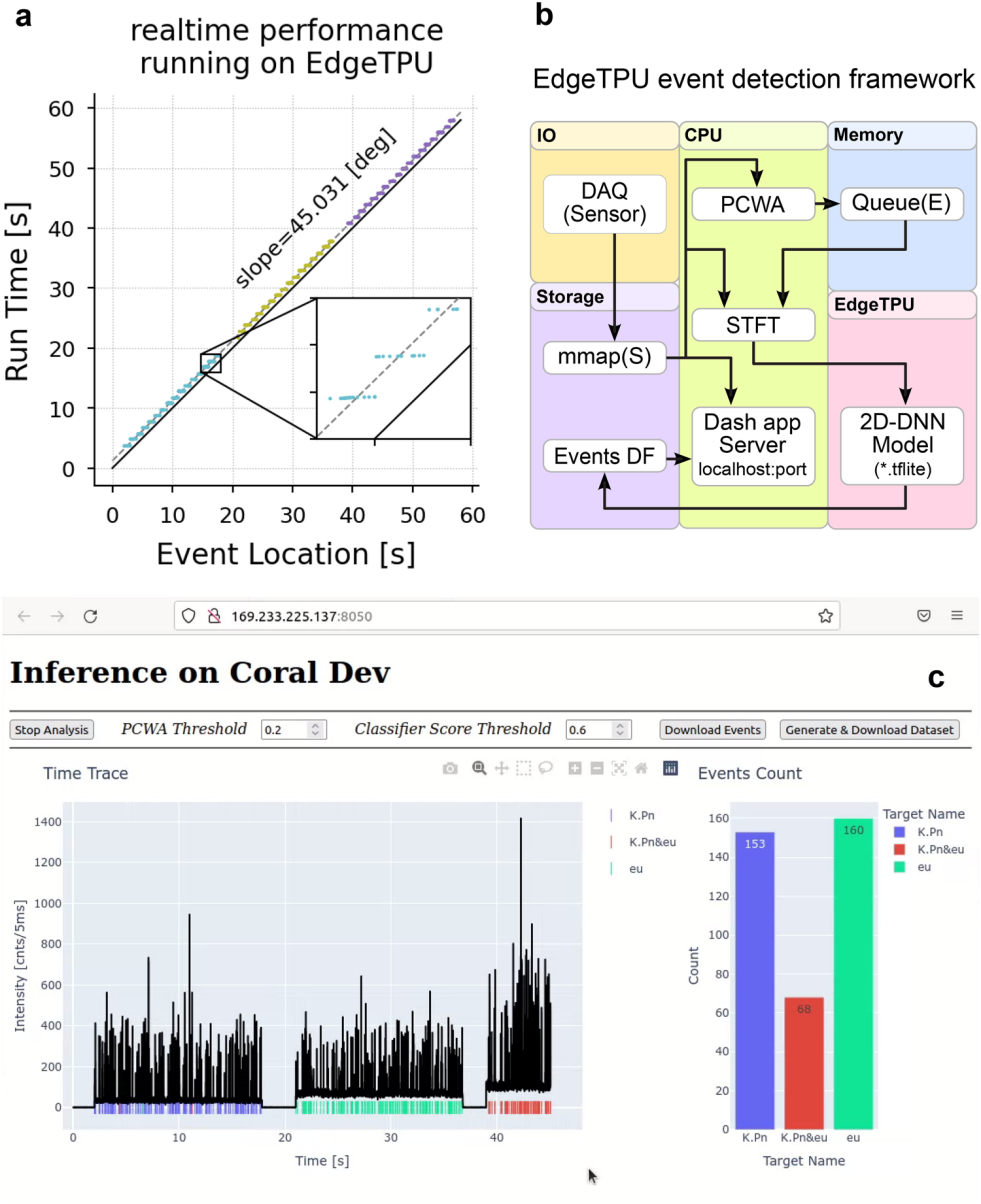
Detection on edge. (**a**) Real-time performance of the model running on Edge-TPU (Coral Dev board). By utilizing a pipelined architecture shown in (**b**), there is only a delay for the first batch, and the rest is processed without any additional delay. The 45-degree slope indicates the real-time calculation of results. The input data is processed in chunks cached in a queue; this is visible in the inset plot where multiple events are revealed at the same time. (**b**) A simplified block diagram showing the essential blocks of the pipelined data processing framework. Some blocks, i.e., DAQ, PCWA, and STFT, run in parallel using Python’s multiprocessing package. Out of 489 detected events on the Edge-TPU, 485 events are confirmed by manual inspection. Of 488 ground-truth events, 485 are correctly located, and 484 are classified, corresponding to 99% accuracy. (**c**) Developed Dash app running on Coral Dev board. Data from the sensor with identified events are visualized in different ways. The dashboard provides good insight into the analyzed sample in real-time.

To create a complete real-time signal analysis system, we designed an event detection and classification framework (Fig. 4b) that integrates sensory data acquisition (DAQ), event detection, preprocessing, DNN inferencing, database management, and visualization processes on a single low-power portable device. The individual blocks are distributed into independent processes and threads to run in parallel, which is necessary to achieve real-time performance with limited resources. Counting individual biomarker targets in real-time can improve diagnostic accuracy by providing better insight into the test right after the start compared to the conventional waiting period and final test result.

The input data trace is first buffered in a queue with fixed-length chunks and then passed to the PCWA event detector process^66^ with a single generic rectangular wavelet (Supplementary Fig. S1b). Locations of detected events are used to crop and send cropped windows from the raw data trace to the STFT block, which then is sent to another queue to be classified by the DNN model. As depicted in Fig. 4a, the Coral Dev Board is capable of identifying events from the fluorescence data trace in real-time. This is the second principal result of this work.

The first batch of events takes tens of milliseconds to process, and due to the pipelined architecture, the remaining batches are processed without any further lag (slope of 45° for run time vs. event time location). Processed batches are visible in the (inset of Fig. 4a) as a group of events returned simultaneously with the same run time tag. Table 1 summarizes the framework’s detection rate and accuracy measures. The detection rate is calculated based on manual inspection of events, and classification is verified by event location in the fluorescence data-trace stage. The input data trace, and location of detected events with additional information, i.e., type of target, velocity, and the classification score, are displayed on a minimal live dashboard. A bar graph is used to display the count of detected events for each target type as the experiment evolves (Fig. 4c). The dashboard runs on a local network without any need for cloud resources. This scheme is an excellent practice for addressing recent data privacy concerns related to cloud-based data processing services^47^.

**Table 1:** Edge-TPU event detection performance.

Measure	Performance (%)
Detection rate	93.8
Classification accuracy	99.8

## Discussion

We have introduced a framework for amplification-free multiplexed, AI-assisted pathogen detection on the fly. The use of a neural network based machine learning algorithm allows for identification and classification of fluorescence signals under realistic, nonideal conditions. Compared to the conventionally used multiplexed single-molecule identification technique, shift-and-multiply, SaM, the classification accuracy for our DNN model has over 40% better ROC-AUC metric while running fast enough to perform classification in real-time. We showed that conventionally demanding data processing tasks such as super high resolution multi-scale event detection and classification can now be done on much smaller devices with a very efficient neural network model containing only a few thousand parameters. Endpoint near-sensor data analysis has proven to be beneficial in terms of energy and network load spent on endpoint-cloud communication plus data privacy and security concerns^47,67^. Our framework solves similar concerns by taking advantage of an AI-specific edge device with an on-demand inferencing strategy. The efficient neural network model runs inference only if an event is detected in the time trace via the PCWA event detection process. Currently, the Coral Dev board is not capable of training the model and requires a desktop computer for training and model transfer steps. We believe this limitation can be solved with future updates of the hardware and libraries or by utilizing a cloud-based server for this specific task. Another current limitation is the upper limit for concentration range; more specifically, the events have to be spaced apart on the time axis. High concentration solutions can be easily diluted with the buffer, with the caveat that dilution will increase sample volume and test times. Future work will tackle this problem in the context of multiplexed detection with sensitivity down to single molecular biomarkers^14,68–70^.

## Materials and methods

### Experimental setup

The ARROW-based fluorescence single-molecule detection setup is depicted in (Fig. 1a). Two lasers running at 738 nm (Ti:Sapphire, Del Mar Photonics) and 556 nm (SSD Nd:YAG, Shanghai Dream Laser Technology Co.) are coupled into a single-mode optical fiber (F-SA, Newport) using a 60× microscope objective (Newport). A pair of modified PC cooling fans are used as mechanical shutters (MS1, MS2) to close/open optical paths for each color. The optofluidic chip is mounted on a 3D printed custom stage using double-sided tape, and the two brass cylindrical fluid reservoirs are glued to the liquid channel ends with wax. The vacuum line is connected to the outlet reservoir to provide negative pressure for sample flow inside the ARROW chip. The fluorescence signal from the labeled targets is guided through the collection waveguide and gathered from the side-facet by a 60× objective (Newport). The excitation light is then removed by a penta-bandpass optical filter (FF01-440/521/607/694/809-25, Semrock) before coupling the collected light with a multi-mode fiber optic patch cable with an FC connector. A single-photon counting module (SPCM-AQRH, Excelitas Technologies) converts fluorescence photons into electrical pulses, and a time-correlated single-photon counting (TCSPC) board (TimeHarp 260 nano, PicoQuant) records time-tagged photon events onto the computer storage disk.

### Sample preparation

10 µL of 3 µM synthetic nucleic acid strands (corresponding to antibiotic-resistant bacterial target) is mixed with 10 µL of 10 µM target-specific fluorescent probe oligomers [IDT DNA Inc.] (see Supplementary Table S1). The final volume for the target-probe mixture is made to 30 µL by adding 1XT50 buffer. The target-probe mixture is heated to 95 °C for 5 min and incubated for 3 h. Streptavidin-coated magnetic beads [DynabeadsTM] with 1 µm diameter are functionalized with target-specific biotinylated capture oligomers. After incubation, the hybridized target-probe structure is mixed with the functionalized magnetic bead in a rotator mixer for 1 h. A magnet kept under the vial is used to pull down the magnetic beads with the captured target-probe complex. All unbound nucleic acid strands are washed off, and the beads are resuspended. Figure 1a (inset) visualizes the final assay structure. This labeling technique has been previously shown to be very specific to the target sequence^14^.

### PCWA event detection and training dataset preparation

The training dataset is created from the time trace shown in Fig. 1b. Events are detected by running the PCWA algorithm with three wavelet functions (MSG-6, MSG-8, and MSG-6&8) to pick three possible classes of events from the time trace. PCWA parameters are set as follows: scales from 0.1 to 1.0 ms with 50 logarithmic steps, spreading coefficients, *h* = 1, *w* = 1, and selectivity = 0.3. PCWA returns time location, scale value, CWT coefficient, and corresponding best-matched wavelet function in the form of a structured array which is then used to crop a window of 1024 datapoint from the 0.01 ms binned time trace centered at events location to form the training dataset. The best-matched wavelet function index is used as the label value in the dataset. An extra step of event quality check (see Supplementary Fig. S2) eliminates overlapping and poorly detected events from the training set.

### Deep neural network model and training

The input signal is converted into a time–frequency spectrogram using short-time-Fourier-transform (STFT) with a 128 datapoint long Hanning window and 120 data points overlapping segments to ensure a balanced time–frequency resolution. A deep neural network with two sets of 2D convolutional layers with ReLU activation functions and 2D max-pooling layers are cascaded after a 2D average-pooling layer on the input image (spectrogram). The average-pooling layer with a pool size of 2 down-samples the input image with a size of 128 × 128 pixels into a 64 × 64 pixels image. We compared the results from the original spectrogram with the down-sampled version, and while we observed a speed up and reduced memory usage during the training and inference, there was no noticeable sacrifice in performance. The first convolutional layer consists of 9 filters with a kernel and stride size of 3 × 3 and 1 × 1 pixels, respectively. An additional batch normalization layer is used prior to the max-pooling layer to improve the training speed and robustness^71^. The second convolutional layer is a stack of 25 5 × 5 pixels filters with the same stride size as the first convolutional layer. Max-pooling layers have pool sizes equal to the kernel sizes used in the corresponding leading convolutional layer. The extracted features with the dimensionality of (3, 3, 25) are then flattened and fed into a dense layer with a softmax activation function which acts as the classifier. The output of the dense layer has three dimensions corresponding to three possible output classes (KPn, eu, and KPneu). A layer of 0.2 dropouts is appended to each convolutional layer as adaptive regularization^72^. The supplementary material presents a detailed DNN model composition (Supplementary Fig. S3a,b). The DNN model is implemented in Python-3.8.10 and TensorFlow-2.8.0 on a 64-bit desktop computer running Ubuntu 20.04.4 LTS.

### Transfer learning and inferencing on the edge

The trained DNN model is quantized into an 8-bit integer model and compiled into an Edge-TPU compatible binary model. A fine-training step involves a few epochs (here 20) of re-exposing the quantized model to the training dataset to recover accuracy from parameters quantization error. The edges-compiler can map nine out of eleven operations to the Edge-TPU, meaning that only input and output float-integer conversions run on the CPU, and the rest of the DNN model operations utilize Edge-TPU resources (see Supplementary Fig. S3c). The compiled model is then transferred into the Coral Dev board. A Python script with blocks shown in (Fig. 4b) is designed to fetch, analyze, and visualize the incoming raw sensory data on the fly. The user interface and dashboard server is implemented using Plotly’s Dash Python library^73^.

## Supplementary Information

Supplementary Information.

## Data Availability

All data that support the findings and conclusions are present in the paper and supplementary materials. Additional data are available from the corresponding author upon reasonable requests.
