# Supplementary material for: Machine learning at the edge for AI-enabled multiplexed pathogen detection
Source: Sci Rep. 2023 Mar 23;13:4744. doi: 10.1038/s41598-023-31694-6 (PMC10034896; doi:10.1038/s41598-023-31694-6)
Supplement: Supplementary file 1 — Supplementary Information. [file 41598_2023_31694_MOESM1_ESM.pdf]

Supplementary Information for Machine learning at the edge for AI-enabled multiplexed pathogen detection

Vahid Ganjalizadeh[1], Gopikrishnan G. Meena[1], Matthew A. Stott[2], Aaron R. Hawkins[2], Holger Schmidt[1,*]

*hschmidt@soe.ucsc.edu

[1]School of Engineering, University of California, Santa Cruz, 1156 High Street, Santa Cruz, CA, 95064, USA.

[2]Electrical and Computer Engineering Department, Brigham Young University, Provo, UT, 84602, USA.

Event detection

The wavelet functions used in the PCWA analysis are shown in Supplementary Fig. S1. Illustrated in Supplementary Fig. S1a, three multi-spot-Gaussian (MSG) wavelets are used to accurately extract location and scale values used in building the training dataset. MSG wavelets are wavelet functions that are custom designed to match the multi-peak signals produced from fluorescence signals[1]. Supplementary Fig. S1b shows a simplified pulse function design to find approximate locations of multi-peak signals. Unlike MSG wavelets, the pulse wavelet is not sensitive to the number, and the combination of peaks does not return a precisely matched scale too. On the Coral Dev Board, a comprehensive deep neural network (DNN) model classifies the events with a minimum score threshold so that any poorly detected event is labeled as unclassified at the end. This single wavelet utilization helps speed up the event detection step while maintaining the accuracy in detection and classification.

Dataset preparation

Detected events are passed through a quality control step where multiple metrics are extracted, as shown in (Supplementary Fig. S2a). Δt is fed by the PCWA algorithm, *W*, and *H* are calculated by fitting a three-segment piecewise function to the normalized cumulative summation (cumsum) of the event signal (Supplementary Fig. S2b) using a differential evolution (DE) optimizer[2]. The signal is 32x down-sampled prior to the cumulative summation step. Extracted features are used to create the scatter plot shown in Supplementary Fig. S2b. The majority of the events sit in the upper-right corner of the plots; therefore, the outliers (overlapping events or events with misleading information) are then easily removed by drawing two threshold lines.

Neural network model

The deep neural network model discussed in the main paper is visualized with more details here. Individual internal layers with information about the output shape and number of parameters can be seen in Supplementary Figs. S3a and S3b. The trained model is then compiled into an Edge-TPU compatible model using the provided edge-tpu-compiler. Nine out of eleven

operations are mapped to run on the Edge-TPU device, and the two remaining operations (quantize and dequantize) run on the ARMx64 CPU on Coral Dev Board (see Supplementary Fig. S3c). Quantize and dequantize layers are necessary to interface data type of 32-bit float into and out of the 8-bit integer Edge-TPU processor. Models are saved in TensorFlow Lite (*.tflite) file format and visualized by Netron webapp[3].

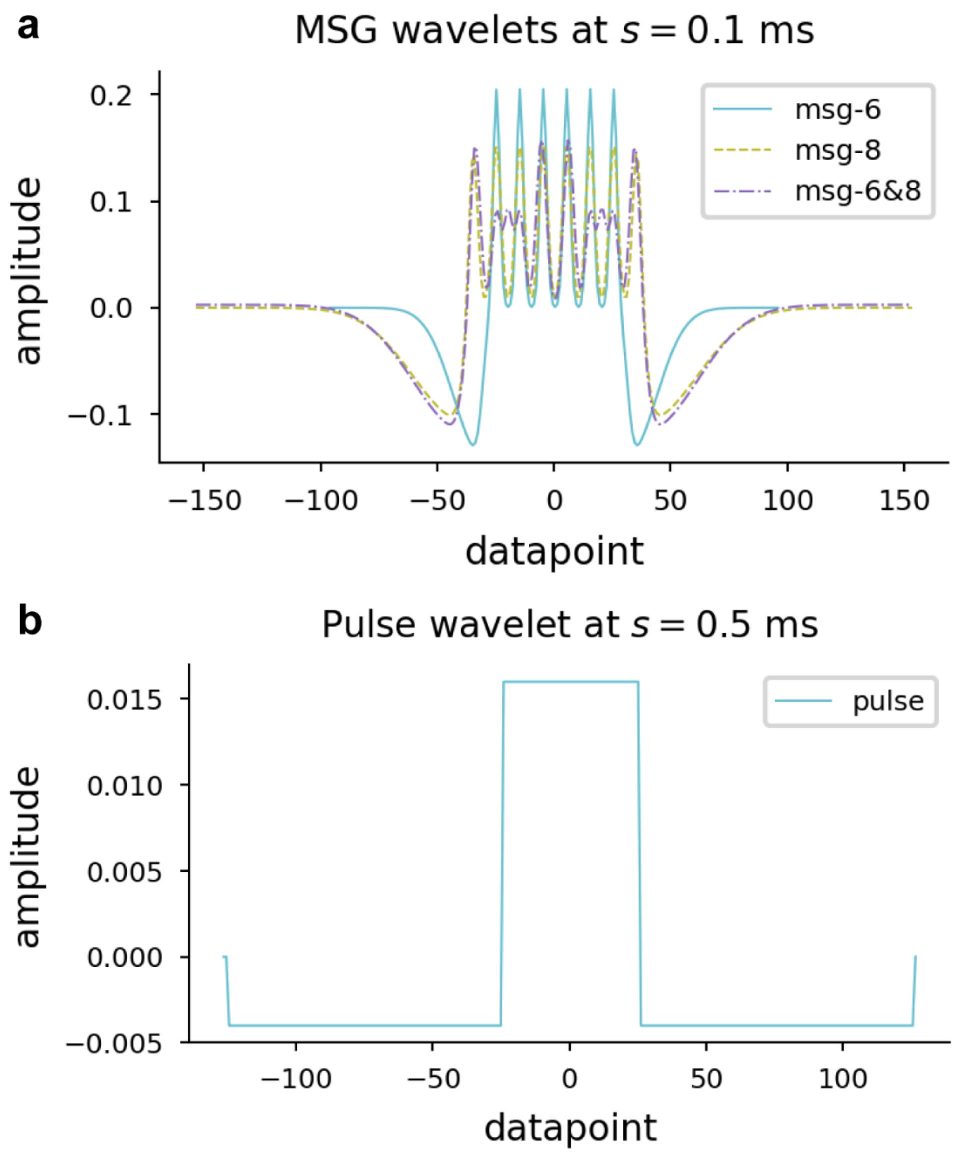

Supplementary Fig. S1 Wavelets used for PCWA event detection algorithm. **a** MSG wavelets at the lowest scale (*s*=0.1 ms). **b** Pulse wavelet at lowest scale (*s*=0.1 ms) used in Coral Dev Board to just detect the approximate location of events.

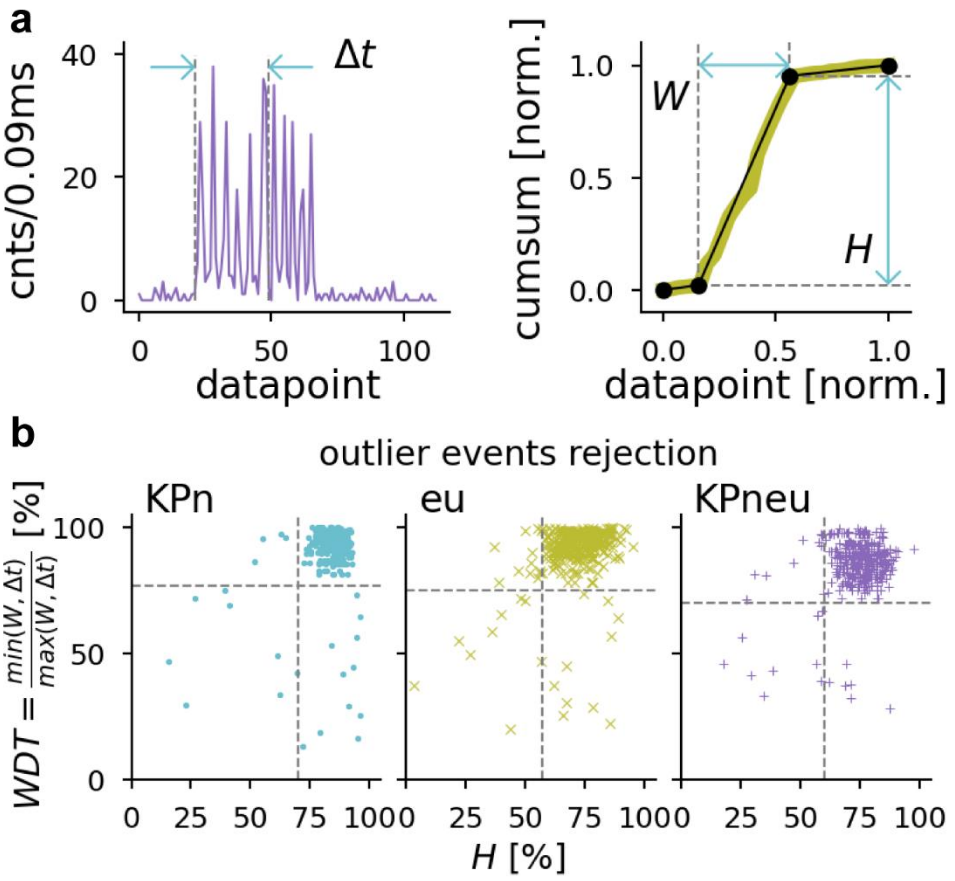

Supplementary Figure S2. Multi-factor signal quality check. **a** On the left is an example of two overlapping KPn events. The extracted Δt parameter from the PCWA analysis is shown. The graph on the right is the normalized cumulative summation of a down-sampled version of the signal on the left. The black line is a 3-segment piecewise function fitted to the cumsum graph using a DE optimizer. The annotated *W* and *H* parameters are extracted for quality check purposes. **b** Scatter plots of extracted quality metrics illustrate how outliers are rejected from the training dataset by setting threshold lines.

a

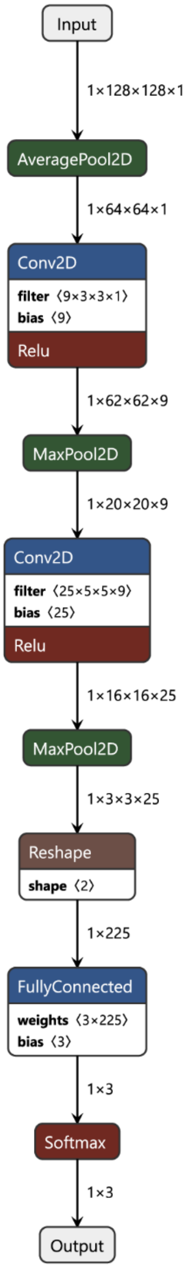

b

Model: "2D_DNN"

Layer (type)	Output Shape	Param #
average_pooling2d (AveragePooling2D)	(None, 64, 64, 1)	0
conv2d (Conv2D)	(None, 62, 62, 9)	90
batch_normalization (BatchNormalization)	(None, 62, 62, 9)	36
activation (Activation)	(None, 62, 62, 9)	0
max_pooling2d (MaxPooling2D)	(None, 20, 20, 9)	0
dropout (Dropout)	(None, 20, 20, 9)	0
conv2d_1 (Conv2D)	(None, 16, 16, 25)	5650
max_pooling2d_1 (MaxPooling2D)	(None, 3, 3, 25)	0
dropout_1 (Dropout)	(None, 3, 3, 25)	0
flatten (Flatten)	(None, 225)	0
dense (Dense)	(None, 3)	678

Total params: 6,454
Trainable params: 6,436
Non-trainable params: 18

c

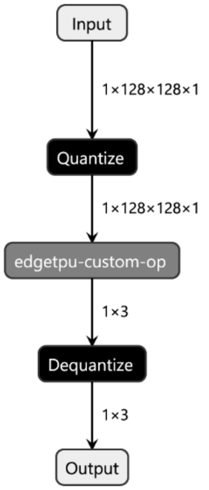

Supplementary Figure S3. 2D-DNN neural network architecture. **a** The visualized graph of internal layers. The batch normalization layer is not displayed by the visualizing application used here. **b** The model summary includes output shapes, and parameters count for individual layers as well as the total parameters. **c** The rendered graph of the compiled model on Edge-TPU. All internal blocks from Supplementary Fig. S3a are mapped to the Edge-TPU processor and shown as a single edgetpu-custom-op block. Two new blocks (Quantize and Dequantize) are added to

convert input/output values from 32-bit float to the 8-bit integer Edge-TPU processor values and vice versa. These two added units run on the CPU.

Sample preparation

In order to enable efficient and highly specific detection of bacterial biomarkers, we use synthetic oligomers corresponding to K. Pneumoniae with a total length of 128bp:

5'GACTACGGTCGTAACCTTGGCGCCCTGTACGACGTGGAAGCCTGGACCGATATGTTCCCGGAATTCGGCGGCGATTCCTCTGCCCAGACCGATAACTTTATGACCAAGCGCGCCAGCGGCCTGGCGAC**3'**

Of this sequence, 31 bp are used to design a matching red fluorescent beacon, 32 bp for a green fluorescent beacon, and 50 bp for a pulldown sequence on the microbeads. The three sections are highlighted in the corresponding colors in the sequence above (yellow: pulldown sequence). The simulated melting temperatures[4] of the target synthetic nucleic acid strands, capture, red, and green probes in 50 mM Na^+ salt solution are 73.7˚C, 75.9˚C, 69.5˚C, and 71.2˚C respectively. During the hybridization steps, we used a higher concentration of the probe in comparison to the number of binding sites on the microbeads (~10x higher) to make sure the final sample contained magnetic beads on which all potentially attached target molecules were labeled with a fluorescent probe.

Target name	Capture oligomer	Fluorescent probe oligomer	Color code
**Klebsiella Pneumoniae**	/5BiotinTEG/GTCGCCAGGCC GCTGGCGCGCTTGGTCATAAA GTTATCGGTCTGGGCAGA3'	/5Alex546N/ CGCCGCCGAATTCCGGGAACAT ATCGGTCCAG3' 5'CGTACAGGGCGCCAAGGTTA CGACCGTAGTC/3AlexF750N/	Green-Red

Supplementary Table S1. Oligomer used for fluorescent tagging of K. Pneumoniae synthetic nucleic acid and binding to biotinylated capture probes.

Supplementary References

1. Ganjalizadeh, V. & Schmidt, H. Fast custom wavelet analysis technique for single molecule detection and identification. (2021) doi:10.5281/zenodo.5794624.
2. Feoktistov, V. *Differential evolution*. (Springer, 2006).
3. Roeder, L. Netron, Visualizer for neural network, deep learning, and machine learning models. (2017) doi:10.5281/zenodo.6158118.
4. Oligo Analyzer. https://www.idtdna.com/calc/analyzer.
